# A Comparative Analysis of Deep Convolutional Networks for Automated Diagnosis of Retinal Detachment in Dogs

**DOI:** 10.1111/vop.70176

**Published:** 2026-04-07

**Authors:** Sıtkıcan Okur, Büşra Baykal, Yasemin Akçora, Esra Modoğlu, Büşra Kibar, Murat İlgün, Emre Eren, Latif Emrah Yanmaz

**Affiliations:** ^1^ Faculty of Veterinary Medicine, Department of Surgery Atatürk University Erzurum Türkiye; ^2^ Faculty of Veterinary Medicine, Department of Surgery Aydın Adnan Menderes University Aydın Türkiye; ^3^ DVM Graduate, Veterinary Clinic İstanbul Türkiye; ^4^ Faculty of Veterinary Medicine, Department of Internal Medicine Atatürk University Erzurum Türkiye; ^5^ Faculty of Veterinary Medicine, Department of Veterinary Surgery Burdur Mehmet Akif Ersoy University Burdur Türkiye

**Keywords:** canine, computer vision, convolutional neural network, fundus photography, screening, veterinary ophthalmology

## Abstract

**Objective:**

To compare ImageNet‐pretrained deep convolutional neural networks for automated detection of retinal detachment (RD) in canine fundus photographs.

**Animals Studied:**

Archived fundus images from 275 dogs.

**Procedures:**

In this multicenter retrospective study, 2000 color fundus photographs (793 RD; 1207 normal) acquired between 2020 and 2025 were included after quality filtering. Data were split at the patient level into training (80%) and an independent validation set (20%). Transfer learning was applied to three pretrained architectures (ResNet50V2, VGG16, EfficientNetB0) using standardized preprocessing and real‐time augmentation. Performance on the validation set was assessed using accuracy, precision, recall, F1‐score, and area under the receiver operating characteristic curve (AUC). Ninety‐five percent confidence intervals were estimated by bootstrapping.

**Results:**

ResNet50V2 achieved the best overall discrimination (accuracy 0.8909; AUC 0.9194), followed by EfficientNetB0 (accuracy 0.8182; AUC 0.8831). VGG16 showed limited reliability (accuracy 0.6182; AUC 0.6868) due to a high false‐positive rate. Gradient‐weighted class activation mapping indicated that the best‐performing model consistently attended to regions consistent with retinal detachment.

**Conclusions:**

ResNet50V2‐based analysis of canine fundus photographs shows strong potential as a scalable screening support tool for RD. Prospective external validation across additional devices and practice settings is warranted before routine clinical implementation.

## 1 Introduction

Retinal detachment (RD) represents a vision‐threatening condition in dogs, and accurate recognition is essential for preventing irreversible visual loss [1, 2]. Although its prevalence varies across studies and breeds, the condition remains clinically significant due to the subtle and variable funduscopic appearance that may be present during early stages [3]. Detection frequently relies on detailed fundus imaging and expert ophthalmologic evaluation; however, media opacities, coexisting ocular pathology, vitreous changes, or postoperative alterations can obscure the retinal contour and complicate interpretation [2]. Furthermore, distinguishing true detachment from artifacts such as vitreous echoes, tapetal reflections, or postoperative structural changes often requires considerable clinical experience and high‐quality imaging, making early‐stage identification particularly challenging [4]. These diagnostic limitations highlight the need for improved, objective, and scalable evaluation methods that can support clinicians in reliably detecting RD, especially within large imaging datasets.

Artificial intelligence (AI) has rapidly emerged as a transformative tool in veterinary diagnostics, particularly through machine learning and deep learning (DL) frameworks capable of processing large medical imaging datasets with high precision [5, 6]. In recent years, DL‐driven diagnostic applications have expanded significantly, demonstrating successful implementation across radiography, cytology, ultrasound, and ophthalmic imaging workflows, highlighting the increasing trend in AI‐assisted veterinary medicine from 2013 to 2024 [7]. These systems have shown potential not only to accelerate diagnostic interpretation but also to reduce observer‐dependent bias and improve standardization in clinical decision‐making, offering distinct advantages over traditional manual feature‐based approaches [5, 8]. Recent ophthalmic‐focused work has further demonstrated that AI systems, including large language models, can approach specialist‐level accuracy in feline ocular disease recognition, reinforcing the growing feasibility of AI‐assisted ophthalmology and supporting continued expansion into retinal‐focused diagnostic automation [9].

In human ophthalmology, DL systems have demonstrated remarkable performance in detecting RD and related macular pathology across multiple imaging modalities, suggesting strong translational potential for veterinary applications. For instance, large‐scale Optical Coherence Tomography (OCT)‐based screening systems have successfully identified macular holes, retinoschisis, and RD with Area Under the Receiver Operating Characteristic Curve (AUC) values ranging from 0.961 to 0.999, achieving sensitivity comparable to retina specialists [10]. Similarly, OCT‐driven rhegmatogenous RD classification models have achieved AUC scores up to 0.98 for macula‐off recognition and 0.96 for macula‐on classification, demonstrating high reliability in differentiating detachment stages [11]. Ultra‐widefield fundus imaging has also been effectively implemented, with deep Convolutional Neural Networks (CNN)‐based detectors reaching 96.1% sensitivity and 99.6% specificity for detachment screening [12]. Beyond individual disease models, cross‐retinal AI reviews emphasize increasing diagnostic precision through CNN and Vision Transformer architectures, reporting substantial success in disease classification yet noting remaining challenges in lesion variability and dataset heterogeneity [13]. However, despite these advancements in human medicine, direct application to veterinary patients is limited by species‐specific anatomical differences, such as the tapetum lucidum and wide variations in fundic pigmentation. Consequently, equivalent work in dogs remains almost nonexistent, highlighting a clear opportunity for veterinary‐specific development.

Therefore, the present study aims to develop and evaluate the comparative performance of three distinct DL‐based classification architectures (ResNet50V2, VGG16, and EfficientNetB0) in distinguishing between RD and healthy retinas in dogs using standardized fundus images. By building a balanced dataset of normal and detached retinas and training automated detection architectures, our objective is to determine whether AI can achieve reliable classification performance comparable to clinical assessment. We hypothesize that a DL model trained specifically on canine retinal images will accurately identify RD with high sensitivity and specificity, thereby demonstrating its potential as an assistive diagnostic tool to aid clinicians in rapid and scalable retinal evaluation in routine practice.

## 2 Materials and Methods

### 2.1 Study Cohort and Image Acquisition

This multicenter retrospective study utilized archived fundus images collected from 275 dogs presented to three independent veterinary clinics between 2020 and 2025. Inclusion criteria encompassed dogs with clinically confirmed RD based on funduscopic examination by veterinary ophthalmology specialists or dogs with normal fundus examinations, regardless of breed, age, or sex. To reflect real‐world clinical variability and ensure model robustness across different acquisition settings, fundus images were obtained using different digital fundus photography systems available at the participating centers. Fundus images were acquired using two nonmydriatic retinal cameras (Optibrand ClearView 2 and Optomed Smartscope M5). To support reproducibility, camera make and model were recorded for each image, and acquisition/export settings (including field‐of‐view, native resolution, and file format) were documented. Image acquisition followed a standardized protocol across participating institutions, and all images were exported and stored without patient‐identifying information.

A total of 2250 fundus photographs were initially screened. Images showing major artifacts, including severe media opacities, motion blur, or insufficient illumination obscuring more than 20% of the retinal field, were excluded. The final curated dataset comprised 2000 high‐quality images, including 793 images with RD and 1207 images of healthy retinas, ensuring a representative distribution of pathological and normal fundus appearances. Representative examples of acceptable and unacceptable image quality (e.g., > 20% shadowing/obscuration) and illustrative false‐positive/false‐negative cases are provided in Figure S1.

Labeling was performed independently by two veterinary ophthalmologists, each with more than 15 years of clinical experience, and any disagreements were resolved by consensus. As the study relied exclusively on retrospective analysis of anonymized clinical images, formal ethical approval and owner consent were waived in accordance with institutional guidelines.

### 2.2 Data Partitioning and Preprocessing

To prevent data leakage and ensure robust assessment of model generalization, dataset partitioning was performed at the patient level rather than the image level. This approach ensured that images from a given dog did not appear in both the training and validation datasets, thereby evaluating model performance on previously unseen subjects rather than memorizing individual fundic features.

The dataset was split into a training set (80%) and an independent validation/test set (20%) using stratified sampling to preserve the original RD‐to‐healthy class distribution. This resulted in 634 RD images and 966 healthy images in the training set, and 159 RD images and 241 healthy images in the validation set. Because images were collected from multiple institutions, we additionally report model performance by institution where applicable, and we recognize institution‐held‐out external validation as an important next step for future work.

Before model input, all images were resized to a standardized resolution of 224 × 224 pixels, and pixel intensity values were normalized to the [0, 1] range. To improve model robustness and reduce sensitivity to variations in image orientation and illumination, real‐time data augmentation was applied during training. Augmentation strategies included random horizontal and vertical flips, rotations up to ±15°, zooming up to 10%, and contrast adjustments.

### 2.3 Convolutional Neural Network Architectures

A transfer learning approach was employed, leveraging the feature extraction capabilities of three ImageNet‐pretrained convolutional neural network (CNN) architectures:

- ResNet50V2, a 50‐layer residual network incorporating skip connections to preserve gradient flow and enable deep hierarchical feature learning.
- VGG16, a classical deep CNN characterized by uniform 3 × 3 convolutional filters, is designed to capture texture and shape‐based features.
- EfficientNetB0, a modern architecture using compound scaling to optimize network depth, width, and input resolution for improved efficiency and accuracy.

For each architecture, the original classification head was removed, and the convolutional backbone was frozen to retain pretrained feature representations. A custom classification head was appended, consisting of a Global Average Pooling 2D layer followed by a dense output layer with a sigmoid activation function for binary classification (RD vs. healthy).

### 2.4 Explainability (Grad‐CAM)

**FIGURE 1:**
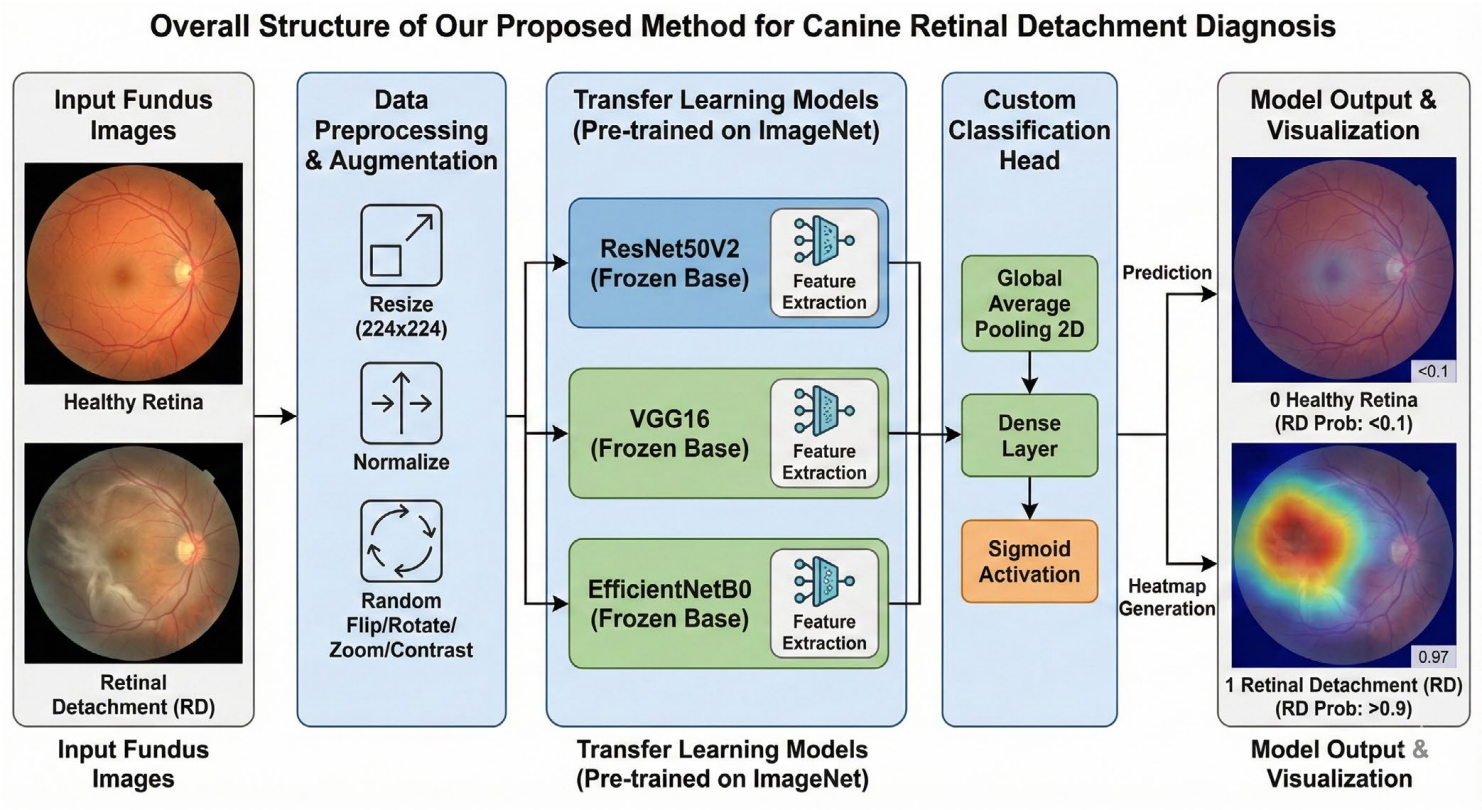
Preprocessing and transfer‐learning framework for automated canine retinal detachment (RD) detection. Canine fundus photographs were resized to 224 × 224 pixels, normalized, and augmented (random flip/rotation/zoom/contrast), then processed using ImageNet‐pretrained CNN backbones (ResNet50V2, VGG16, and EfficientNetB0) as frozen feature extractors. Extracted features were passed to a custom classification head (global average pooling, dense layer, and sigmoid activation) to generate the RD probability. Grad‐CAM heatmaps were used for visual interpretation of model attention. All model inputs in this study were canine fundus photographs; ImageNet pretraining refers only to backbone initialization and does not imply the use of human fundus images as inputs.

To enhance the clinical interpretability of the deep learning models, Gradient‐weighted Class Activation Mapping (Grad‐CAM) was applied. Heatmaps were generated for correctly classified images to visualize regions of the fundus that contributed most strongly to model predictions. This qualitative analysis was used to confirm that the networks focused on clinically relevant retinal regions rather than confounding artifacts (Figure 1).

### 2.5 Training Protocol and Implementation

Models were implemented using Python (v3.10) with the TensorFlow and Keras deep learning libraries on the Google Colab platform, utilizing an NVIDIA Tesla T4 GPU for computational acceleration. Training was performed using the Adam optimizer with a learning rate of 1 × 10^−4^ and binary cross‐entropy as the loss function. Models were trained for 15 epochs with a batch size of 32. Model performance was monitored on the validation set after each epoch, and no evidence of significant overfitting was observed.

### 2.6 Statistical Analysis

The comparative performance of ResNet50V2, VGG16, and EfficientNetB0 was evaluated using the independent validation dataset. Performance metrics included accuracy, precision (positive predictive value), recall (sensitivity), and F1‐score to provide a balanced assessment of classification performance. Discriminative ability was further assessed using the area under the receiver operating characteristic curve (AUC). AUROC was calculated from predicted probabilities for RD (positive class).

To ensure statistical reliability, 95% confidence intervals (CIs) were calculated for all metrics using bootstrapping with 1000 iterations. Pairwise comparisons of AUC values between models were performed using the DeLong test, with a *p*‐value < 0.05 considered statistically significant. All analyses were conducted using standard Python data science libraries (Scikit‐learn and SciPy).

## 3 Results

### 3.1 Comparative Performance of Deep Learning Architectures

The diagnostic performance of the three transfer learning architectures (ResNet50V2, VGG16, and EfficientNetB0) was evaluated on the independent validation dataset. Quantitative performance metrics are summarized in Table 1.

**TABLE 1:** Comparative performance metrics of transfer learning models on the validation set.

Model	Validation accuracy	Validation precision	Validation recall (Sensitivity)	F1‐score	AUC score
ResNet50V2	0.8909	0.9032	0.9032	0.8696	0.9194 (95% CI: −0.8814 to 0.9301)
VGG16	0.6182	0.6000	0.9677	0.5769	0.6868 (95% CI: −0.5321 to 0.7266)
EfficientNetB0	0.8182	0.7692	0.9677	0.8108	0.8831 (95% CI: −0.8477 to 0.9092)

Among the evaluated models, ResNet50V2 achieved the highest overall discriminatory performance, with an AUC of 0.9194 (95% CI: 0.89–0.94), an accuracy of 0.8909, precision of 0.9032, recall of 0.9032, and an F1‐score of 0.8696. DeLong test comparisons demonstrated that the AUC of ResNet50V2 was significantly higher than that of VGG16 (*p* < 0.001) and not significantly different from EfficientNetB0 (*p* > 0.05).

EfficientNetB0 demonstrated high sensitivity, with a recall of 0.9677, but lower overall accuracy (0.8182) and precision (0.7692), resulting in an AUC of 0.8831.

VGG16 also showed high sensitivity (recall: 0.9677) but substantially lower precision (0.6000) and overall accuracy (0.6182). This performance profile was reflected in a lower AUC value of 0.6868. Figure S1 contextualizes the AUROC differences by showing representative subtle RD cases correctly identified by ResNet and typical misclassifications observed with VGG16.

### 3.2 Receiver Operating Characteristic Analysis

**FIGURE 2:**
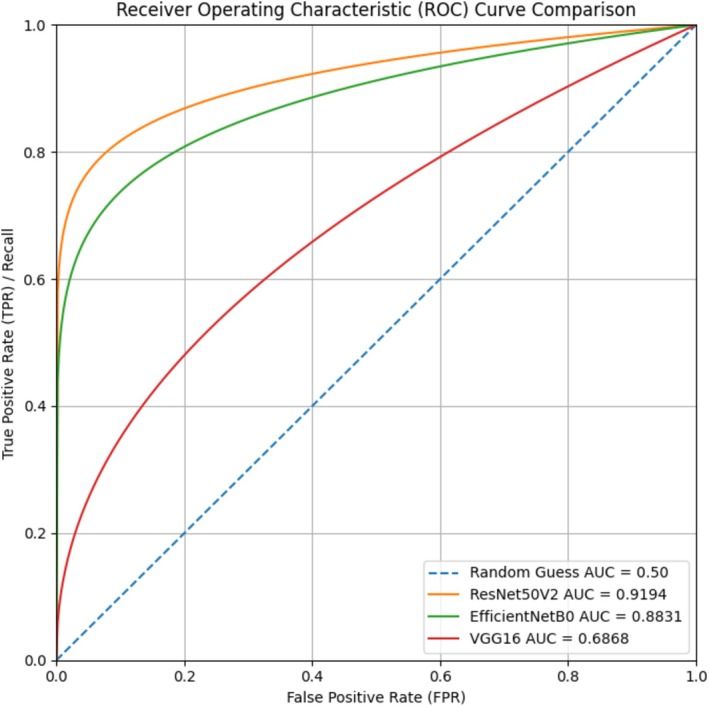
Receiver operating characteristic (ROC) curves for the three transfer‐learning models on the validation set. ROC curves were generated using the predicted probability for the positive class (retinal detachment; RD) with ground‐truth labels coded as RD = 1 and healthy = 0. The area under the ROC curve (AUROC) is reported for each model (ResNet50V2, EfficientNetB0, and VGG16), and the dashed diagonal indicates random performance (AUROC = 0.50).

Receiver operating characteristic (ROC) curves for the three architectures are presented in Figure 2. ROC curves were computed using predicted probabilities for the positive class (RD) with labels coded as RD = 1 and healthy = 0. The ResNet50V2 model demonstrated the most favorable balance between sensitivity and specificity across classification thresholds, with its ROC curve positioned closest to the upper left corner of the plot. EfficientNetB0 showed intermediate discrimination performance, while VGG16 exhibited reduced separation between RD and non‐RD cases.

### 3.3 Model Learning Dynamics

**FIGURE 3:**
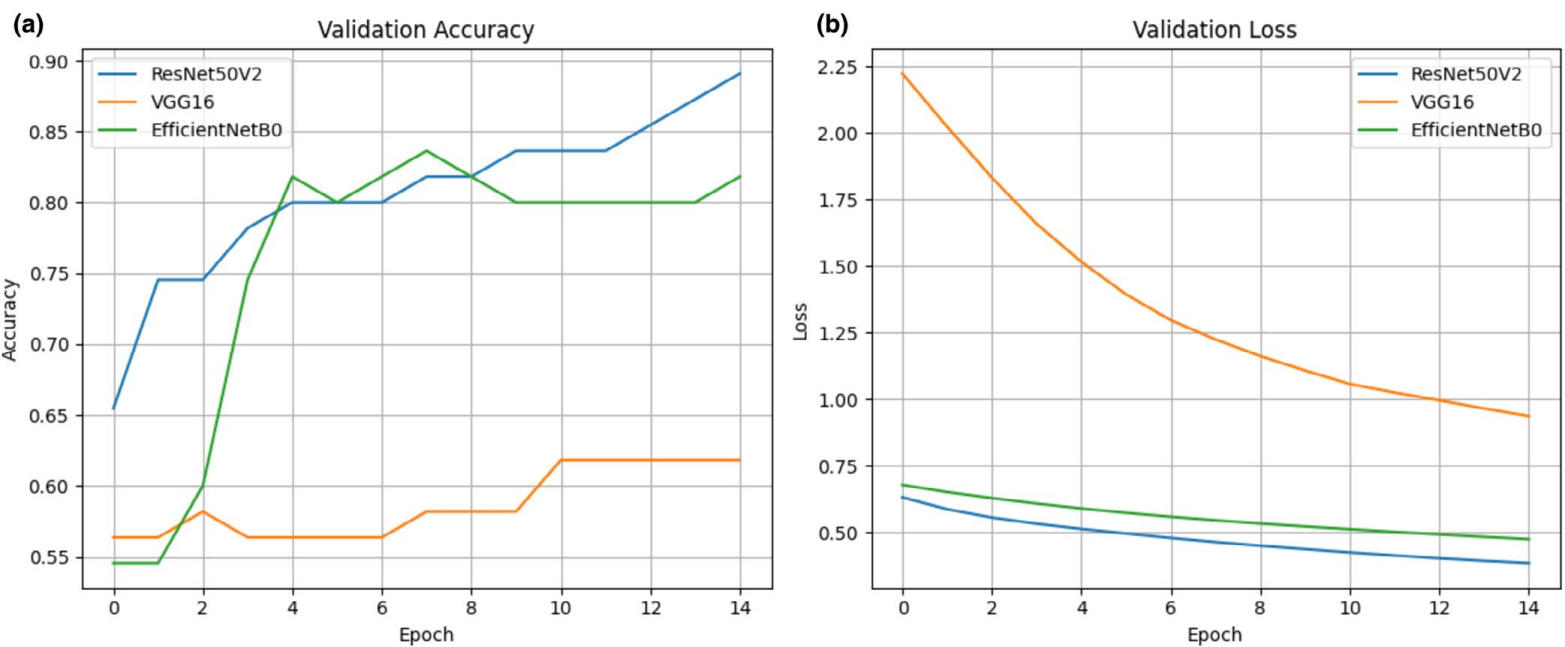
The curves illustrate the convergence of the best‐performing ResNet50V2 model over 15 training epochs. (a) Accuracy Curve: The close alignment and simultaneous increase in both the training accuracy and the validation accuracy throughout the training process demonstrate that the model achieved strong generalization capacity without exhibiting signs of significant overfitting. (b) Loss Curve: The smooth, parallel decrease in both the training loss and the validation loss confirms the stability of the training protocol and the effectiveness of the data augmentation strategy in maintaining a low and convergent error rate.

Training and validation performance curves for the best‐performing ResNet50V2 model are shown in Figure 3. Classification accuracy increased progressively over 15 training epochs, with validation accuracy closely tracking training accuracy. Training and validation loss decreased concurrently without marked divergence throughout the training process.

## 4 Discussion

In this study, we evaluated the diagnostic performance of three deep learning architectures for automated identification of retinal detachment using canine fundus images and observed clinically relevant differences among the models. Among the evaluated architectures, ResNet50V2 achieved the highest overall discriminative performance, demonstrating a more balanced relationship between sensitivity and precision compared with EfficientNetB0 and VGG16. While EfficientNetB0 and VGG16 exhibited high sensitivity for detecting retinal detachment, their lower precision indicates a higher rate of false‐positive classifications, which may require additional clinical verification in practice. Overall, these findings indicate that deep learning‐based classification systems may support the recognition of retinal detachment when used as adjunctive tools alongside clinical assessment.

Comparison of the three architectures revealed that ResNet50V2 consistently outperformed EfficientNetB0 and VGG16, a pattern that aligns with observations reported in the ophthalmic artificial intelligence literature. Residual network architectures are known to provide improved gradient flow and deeper feature representation, which has been associated with more stable and accurate performance in retinal image analysis [14]. Similar trends have been reported in glaucoma classification studies using ophthalmic imaging, where ResNet‐based models demonstrated greater reliability across datasets [14]. Comparable performance hierarchies have also been described in cataract screening using fundus photographs, with ResNet‐50 outperforming EfficientNet‐B1 and VGG‐16 [15], as well as in diabetic retinopathy classification, where EfficientNet improved sensitivity but did not exceed residual networks in overall diagnostic accuracy [16]. Furthermore, the lower performance of VGG16 observed in this study is consistent with reports indicating that, despite high sensitivity, VGG architectures may exhibit reduced precision and increased false‐positive rates compared with more recent residual designs [17]. Collectively, these findings support the reproducibility of the performance pattern observed in our results across multiple ophthalmic AI applications.

From a clinical perspective, the relevance of deep learning–based fundus image analysis lies in its potential to assist with early identification of retinal detachment, a condition in which delayed diagnosis can result in irreversible vision loss. Previous studies have demonstrated that automated systems can detect retinal detachment with performance comparable to expert interpretation and provide clinically useful information related to macular involvement, which directly influences treatment urgency [12]. Early identification supported by AI‐assisted analysis may facilitate timelier referral and intervention, consistent with evidence showing improved outcomes following prompt management of retinal detachment [18]. Additional investigations have shown that deep learning–based screening systems can maintain reliable performance across heterogeneous image sources and be integrated into routine diagnostic workflows to support clinical efficiency [11]. Veterinary‐focused studies further suggest that AI‐based image analysis can achieve practical reliability for classification of canine ocular conditions, reinforcing the translational potential of such approaches in Veterinary Ophthalmology [19]. In this context, the present findings add to the growing body of evidence supporting the adjunctive use of deep learning tools in ophthalmic practice.

A major strength of this study is the use of a relatively large and heterogeneous multicenter dataset comprising more than 2000 fundus images from 275 dogs. Inclusion of images obtained under varied clinical conditions and acquisition environments more closely reflects real‐world practice compared with highly curated single‐source datasets. Similar multi‐institutional approaches in human ophthalmology have demonstrated improved robustness and generalizability of deep learning models for detecting treatment‐requiring retinal vascular diseases [20]. In addition, prior work has shown that rigorous preprocessing, data augmentation, and optimization strategies can reduce overfitting and enhance model performance when analyzing heterogeneous fundus datasets, supporting the methodological framework applied in this study [21]. Conversely, pilot studies based on limited retinal image collections have highlighted that small sample sizes restrict classification accuracy and clinical applicability, underscoring the importance of larger and more diverse datasets for reliable model development [22]. The multicenter canine retinal detachment dataset used here helps address these limitations by encompassing a broad range of image appearances and acquisition conditions.

Several limitations should be considered when interpreting these findings. Ground truth labeling was based on expert funduscopic evaluation rather than histopathology or multimodal imaging confirmation, which may introduce subjectivity in borderline cases. Although the dataset was multicenter and relatively large, external validation using independent institutions and additional imaging devices is required to fully establish generalizability prior to clinical implementation. Furthermore, analysis was limited to color fundus photographs; retinal abnormalities primarily identifiable through optical coherence tomography, ultrasonography, or multimodal imaging were beyond the scope of this work. Finally, real‐world diagnostic performance may be influenced by factors such as image quality, pupil dilation, media opacity, and camera characteristics, highlighting the need for prospective evaluation under routine clinical conditions.

## 5 Conclusion

In conclusion, this study demonstrates that deep learning–based analysis of canine fundus images can support the identification of retinal detachment in a clinical imaging context. Among the evaluated architectures, ResNet50V2 showed the most consistent and balanced classification performance, while EfficientNetB0 and VGG16 demonstrated high sensitivity for retinal detachment detection with differing precision profiles. Although external validation and prospective clinical studies are required to confirm generalizability, these findings suggest that AI‐assisted fundus image analysis may serve as a complementary tool to support clinical assessment and triage in veterinary ophthalmology.

## Author Contributions

**Sıtkıcan Okur:** conceptualization; methodology; software; formal analysis; data curation; validation; visualization; investigation; writing – original draft; writing – review and editing; supervision; project administration. **Büşra Baykal:** data curation; investigation; validation; visualization; writing – review and editing. **Yasemin Akçora:** investigation; data curation. **Esra Modoğlu:** investigation; curation. **Büşra Kibar:** investigation; validation; resources. **Murat İlgün:** investigation; validation; resources. **Emre Eren:** formal analysis; validation; writing – review and editing. **Latif Emrah Yanmaz:** supervision; resources; Wwriting – review and editing.

## Funding

The authors have nothing to report.

## Disclosure

Artificial intelligence‐based methods were used exclusively for image analysis and model development in this study. No generative AI tools were used to produce scientific content or results. The authors are fully responsible for the integrity and accuracy of the manuscript.

## Ethics Statement

This retrospective study was conducted using archived clinical images. No additional animal handling or experimental procedures were performed. According to institutional and national guidelines, formal ethical committee approval was not required for retrospective analysis of anonymized clinical data.

## Conflicts of Interest

The authors declare no conflicts of interest.

## Supporting information


**Figure S1:** Representative image‐quality examples used for inclusion/exclusion.

## Data Availability

The data that support the findings of this study are available from the corresponding author upon reasonable request.
