# Supplementary figures and images for: A Comparative Analysis of Deep Convolutional Networks for Automated Diagnosis of Retinal Detachment in Dogs
Source: Vet Ophthalmol. 2026 Apr 7;29(3):e70176. doi: 10.1111/vop.70176 (PMC13055114; doi:10.1111/vop.70176)

**Supplementary Figure S1**


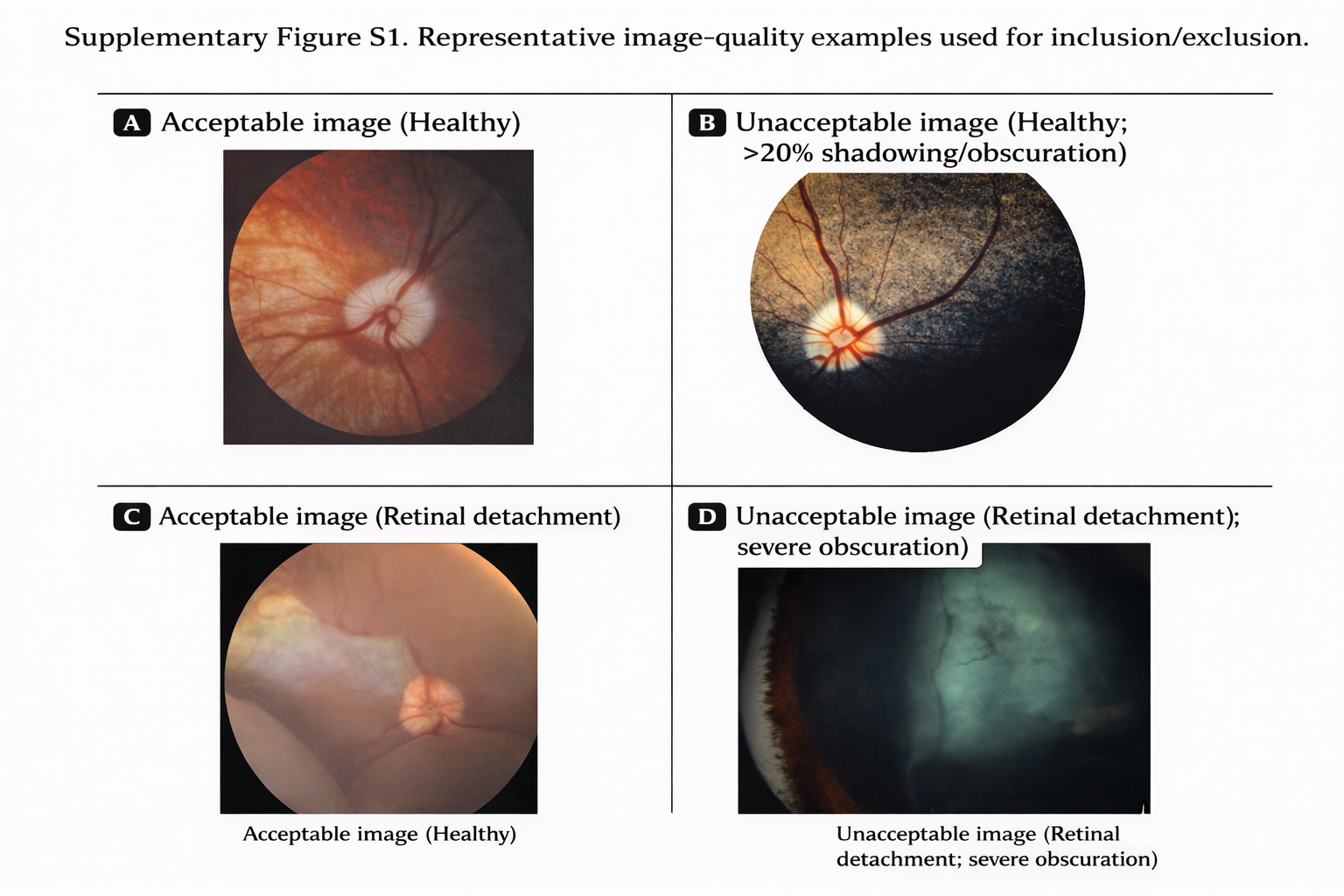

Supplement: Supplementary file 1 — Figure S1: Representative image‐quality examples used for inclusion/exclusion. [file VOP-29-0-s001.docx]
